# The many layers of BOLD. The effect of hypercapnic and hyperoxic stimuli on macro- and micro-vascular compartments quantified by *CVR*, *M*, and *CBV* across cortical depth

**DOI:** 10.1177/0271678X221133972

**Published:** 2022-10-19

**Authors:** Wouter Schellekens, Alex A Bhogal, Emiel CA Roefs, Mario G Báez-Yáñez, Jeroen CW Siero, Natalia Petridou

**Affiliations:** 1 Radiology Department, Center for Image Sciences, UMC Utrecht, Netherlands; 2 Spinoza Centre for Neuroimaging, Amsterdam, The Netherlands

**Keywords:** Laminar fMRI, vasculature, hypercapnia, hyperoxia, spin-echo

## Abstract

Ultra-high field functional magnetic resonance imaging (fMRI) offers the spatial resolution to measure neuronal activity at the scale of cortical layers. However, cortical depth dependent vascularization differences, such as a higher prevalence of macro-vascular compartments near the pial surface, have a confounding effect on depth-resolved blood-oxygen-level dependent (BOLD) fMRI signals. In the current study, we use hypercapnic and hyperoxic breathing conditions to quantify the influence of all venous vascular and micro-vascular compartments on laminar BOLD fMRI, as measured with gradient-echo (GE) and spin-echo (SE) scan sequences, respectively. We find that all venous vascular and micro-vascular compartments are capable of comparable theoretical maximum signal intensities, as represented by the M-value parameter. However, the capacity for vessel dilation, as reflected by the cerebrovascular reactivity (CVR), is approximately two and a half times larger for all venous vascular compartments combined compared to the micro-vasculature at superficial layers. Finally, there is roughly a 35% difference in estimates of CBV changes between all venous vascular and micro-vascular compartments, although this relative difference was approximately uniform across cortical depth. Thus, our results suggest that fMRI BOLD signal differences across cortical depth are likely caused by differences in dilation properties between macro- and micro-vascular compartments.

## Introduction

The human cortex is made up of several different layers. These layers can be distinguished on the basis of different neuronal cell types, as well as their connections with other cortical areas or sensory organs.^1^ Consequently, the different cortical layers are hypothesized to account for different sub-processes in brain functioning and human behavior at large.^2^ Functional magnetic resonance imaging (fMRI) is one of the most powerful tools for studying brain function in both healthy and diseased individuals non-invasively. Recent advances in ultra-high field MRI (i.e., magnetic field strength ≥7 tesla) allow for the investigation of layer-specific neuronal activity in humans.^3,4^ The majority of fMRI studies use the blood-oxygen-level dependent (BOLD) contrast to investigate neuronal functions due to its superior sensitivity.^5^ However, the BOLD signal is an indirect measure of neuronal activity, as the signal primarily originates from differences in the ratio of oxy-hemoglobin [Hb] and deoxy-hemoglobin [dHb], which can be observed through T2 and T2* relaxation times. The change in [Hb]/[dHb] ratio following neuronal activity is mainly caused by increases in cerebral blood flow (CBF) and cerebral blood volume (CBV), resulting from vessel dilation in the capillary beds, venules, and larger veins, as well as increases in the cerebral metabolic rate of oxygen (CMRO_2_).^6,7^

Despite the indirect representation of neuronal activity, BOLD fMRI is known to correspond well with neuronal electrophysiological recordings (local field potentials (LFP) in particular) in both animals and humans.^8–10^ Thus, different vascular characteristics between differently sized venous vascular compartments (e.g., CBF or CBV differences between capillaries, venules and veins) do not prevent the utilization of the BOLD signal as an adequate proxy for neuronal activation in conventional fMRI studies. However, fMRI measurements at the spatial resolution of cortical layers (i.e., depth-resolved, or laminar fMRI) are affected by the vasculature at a different level. The vascular architecture changes across cortical depth, which introduces a confounding correlation between different vascular compartments and the different cortical layers.^11,12^ This is particularly problematic for the most commonly used fMRI acquisition sequence: gradient-echo (GE) BOLD. GE BOLD is sensitive to all venous vascular compartments (i.e., capillaries, venules, veins), but the signal magnitude scales with vessel diameter.^13–17^ GE BOLD, therefore, is disproportionally sensitive to larger (draining) veins, which predominantly reside near the cortical pial surface. This hypersensitivity to macro-vascular compartments leads to an increase in BOLD signal responses measured at superficial layers, while simultaneously suffering from a decrease in specificity to the true site of neuronal activation, since the largest veins pool blood from extended regions of cortex.^18^ Therefore, even the normalization of the raw GE BOLD signal (i.e., to % signal change), cannot compensate for the fact that neuronal populations of different sizes, represented through different vascular compartments, contribute to the BOLD signal differently across cortical depth. The field of laminar fMRI is currently lacking a quantification of the effect of different vascular compartments on the BOLD signal at the laminar level. Here, we address this topic by conducting fMRI measurements in which we record from macro- and micro-vascular compartments across cortical depth, while applying vasoactive stimuli to characterize the confounding correlation between differently sized vascular compartments and the BOLD signal.

We capitalize on the increased BOLD contrast-to-noise ratio (CNR) afforded at 7 tesla along with boosted sensitivity obtained using a high-density surface receive array^19^ to acquire GE and spin-echo (SE) BOLD fMRI measurements at different cortical depths. Where the GE BOLD signal magnitude is skewed towards the macro-vasculature, the SE BOLD signal is generally believed to reflect signals originating from the micro-vasculature (i.e., mostly capillaries and smaller arterioles and venules) at high field strengths.^16,20,21^ Unlike the macro-vasculature, the micro-vasculature distribution is approximately uniform across cortical depth, and is not believed to be capable of vessel dilation in a similar fashion as larger veins.^11,12,22^ To characterize the effects of vasoactive stimuli on macro- and micro-vascular compartments, we manipulate the CBF and CBV by increasing the arterial pressure of CO_2_, a potent vasodilator, in a controlled manner using a computer-controlled gas delivery system.^23–25^ Increases in CBF and CBV decrease the relative venous [dHb] content, which leads to a BOLD signal increase. Administration of an hyperoxic stimulus by increasing the inhaled concentration of O_2_ causes a relative increase in the venous concentration of [Hb] without corresponding vessel dilation, which also leads to a BOLD signal increase. Besides the estimation of the BOLD signal change as a result of vasoactive stimulation, the hypercapnia and hyperoxia breathing conditions were used to estimate changes in cerebral vascular reactivity (CVR), which represents the capacity for vessel dilation;^26–28^ the M-value, reflective of the theoretical maximal BOLD signal change;^29,30^ and the change in relative CBV during separate levels of hypercapnia.^24,31^ With these parameters, we can quantify to what extent the amplitude of the BOLD response is caused by a vessel’s capacity for dilation (CVR), the maximum venous oxygen content (M-value), and the relative CBV increase.

We expect a BOLD signal increase (%ΔBOLD) for all vascular compartments as sampled differently by GE and SE BOLD, during both hypercapnic and hyperoxic breathing conditions across cortical depth. However, the %ΔBOLD as well as CVR, M-value, and ΔCBV sampled from all venous cortical vessels (GE BOLD) are hypothesized to increase from deep to superficial layer estimates, but not for micro-vascular compartments (SE BOLD). Finally, vasculature-dependent ratios for CVR, M-value, and ΔCBV are calculated, describing the effective relative contribution of these metrics observed in different vascular compartments to laminar BOLD fMRI.

## Methods

### Participants

Eleven healthy volunteers (N = 11, age range 18–42 y, mean age = 24.3 y, Female = 8) participated in this study after giving written informed consent. All participants declared that they did not experience breathing difficulties under normal conditions and had not been diagnosed with (cerebro)vascular-related illnesses. The experimental protocol was approved by the local ethics committee of the University Medical Center Utrecht (UMCU) in accordance with the Declaration of Helsinki (2013) and the Dutch Medical Research Involving Human Subjects Act.

### Breathing protocol

**Figure 1.**
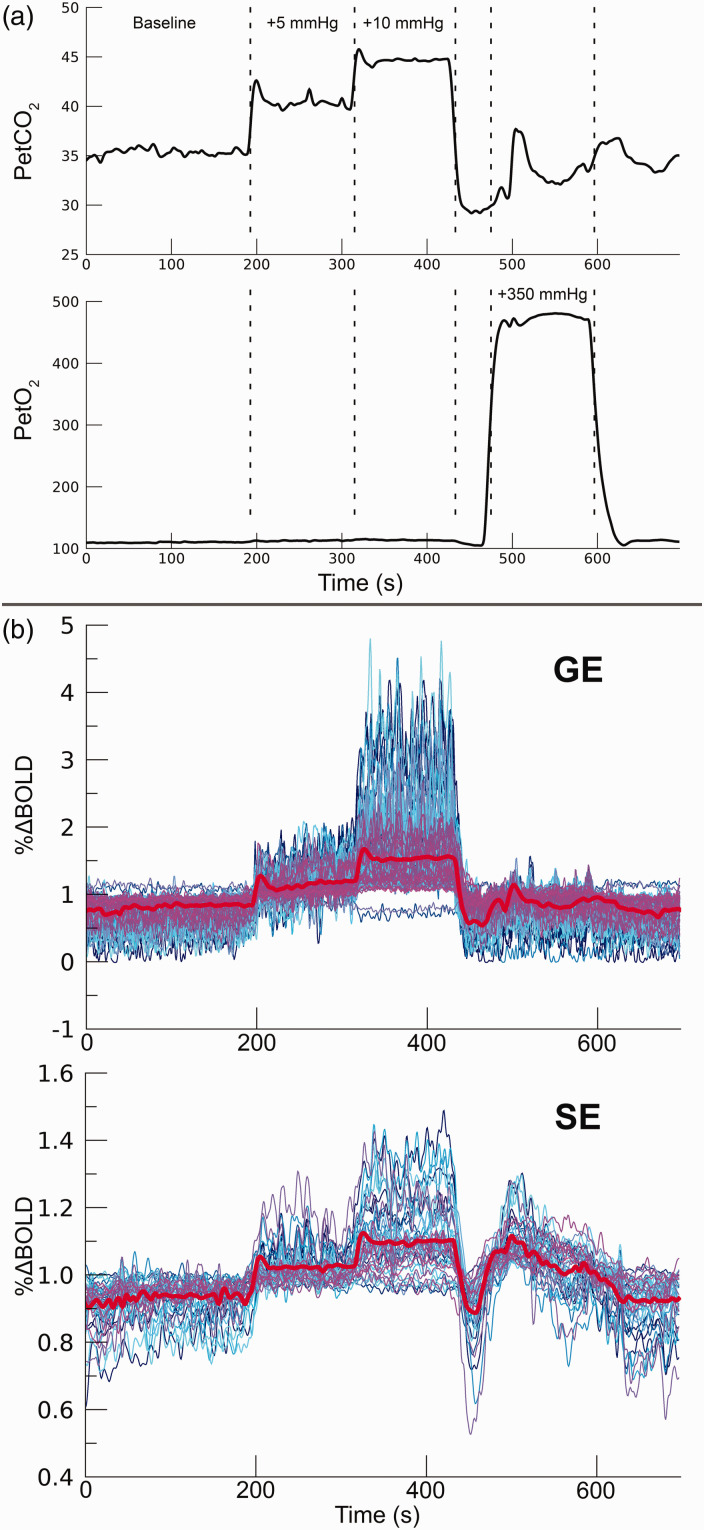
Breathing protocol. a) For 1 participant (subj08) the RespirAct™ measured breathing traces are shown for PetCO_2_ (top panel) and PetO_2_ (bottom panel). The dashed lines depict the hypercapnic conditions (+5 mmHg & +10 mmHg PetCO_2_), and the hyperoxic condition (+350 mmHg PetO_2_). For 7 participants the experiment was repeated with the +5 mmHg and +10 mmHg PetCO_2_ hypercapnic conditions replaced by +3 mmHg and +8 mmHg PetCO_2_, respectively. b) functional timeseries (subj08) of 100 best voxels on the basis of t-statistics from GE BOLD (top panel) and 50 best voxels from SE BOLD (bottom panel). This particular set of voxels is selected for display purposes only, while the analysis was performed on the selection of statistically significant voxels (see also Statistical analysis). The mean timeseries is depicted by the thick red line.

During the acquisition of the functional BOLD time-series (see details below), we administered specific breathable gas mixtures to the participants. Hypercapnia and hyperoxia conditions were achieved by increasing the CO_2_ and O_2_ gas concentrations, respectively. Partial pressure end-tidal (Pet)CO_2_ and PetO_2_ values were targeted using a computer-controlled gas blender and sequential gas delivery system. (3rd generation RespirAct™, Thornhill Research Inc, Toronto, Canada). A 697 s breathing task was performed consisting of the following 4 parts: (1) 200 s baseline period with participant-specific targeted PetCO_2_ values. (2) 120 s hypercapnia period of +5 mmHg PetCO_2_ increase. (3) 120 s hypercapnia period of +10 mmHg PetCO_2_ increase. (4) 120 s hyperoxia period of +350 mmHg PetO_2_ increase (Figure 1(a)). The breathing task was performed twice by all participants: once for each scan acquisition sequence (i.e., GE and SE). For several participants, the experiments were repeated during the same session with +5 mmHg and +10 mmHg PetCO_2_ hypercapnia conditions replaced by +3 mmHg and +8 mmHg PetCO_2_ increases (Supplementary material I). The participant-specific baseline PetCO_2_ calibration was estimated before scanning. Finally, participants were also shown brief visual stimuli (200 ms) that were presented at random intervals (mean frequency of stimulus presentation was 0.1 Hz) throughout the entire task. The results of the visual stimulus were not analyzed here, and only the effects of the vasoactive stimuli are reported hereafter.

### Scan protocol

Scanning was performed using a 7T Philips Achieva scanner (Philips Healthcare, Best, the Netherlands) with two 16-channel high-density surface receive arrays.^19^ Anatomical scans consisted of a T1-weighted volume: MPRAGE with field of view (FOV): anterior-posterior (AP) × inferior-superior (IS) × right-left (RL) = 40 × 159 × 159 mm^3^, which covered the posterior part of the brain (occipital lobe/early visual cortex), voxel size: of 0.8 × 0.8 × 0.8 mm^3^, and TR/TE = 7.0/2.97 ms. T1-weighted volumes at high field strength can experience substantial intensity inhomogeneities. Therefore, a proton density (PD) volume of equal dimensions was acquired to correct for these large-scale intensity inhomogeneities. Finally, three T2*-weighted flow-compensated anatomical volumes were acquired with similar coverage using 3 D-EPI^32^: FOV AP × IS × RL = 40 × 161 × 161 mm^3^ and 0.5 × 0.5 ×0.5 mm^3^ voxel size, TR/TE = 56/30 ms. Both magnitude and phase volumes were reconstructed.

Functional volumes were acquired with GE and SE echo planar imaging (EPI). The GE volumes were acquired with SENSE-factor = 4.0, EPI-factor = 31, water-fat chemical shift = 30 pixels, TR/TE = 850/27 ms, flip-angle (FA) = 50°, voxel size = 1.0 × 1.0 ×1.0 mm^3^, FOV AP × IS × RL = 7 × 128 × 128 mm^3^, covering a portion of early visual cortex within the occipital lobe. The SE volumes were acquired with the following parameters: SENSE-factor = 2.0, EPI-factor = 63, water-fat chemical shift = 45 pixels, TR/TE = 850/50 ms, FA = 90°, voxel size = 1.5 × 1.5 ×1.5 mm^3^, FOV AP × IS × RL = 7.5 × 190 × 190 mm^3^. A lower spatial resolution for SE volumes was chosen to increase the signal-to-noise ratio (SNR), approximating the SNR of our GE volumes, while maintaining settings for SENSE-factor^33^ and water-fat chemical shift^34^ within acceptable ranges. During a single session, a maximum of four fMRI time series (i.e., 2 × GE and 2 × SE) were recorded, during which the different breathing protocols were applied. Each time series consisted of 820 volumes (duration = 697 s per time series, Figure 1(b)). For both GE and SE sequences, 5 volumes with reversed phase encoding were acquired to correct for geometric distortions. During all acquisitions, respiration was measured with a respiratory belt around the chest, and blood pulsation with a peripheral pulse unit (PPU). The respiration and PPU measurements were used to calculate the respiration volume per time (RVT) and beats per minute (BPM).^35^

### Preprocessing

**Figure 2.**
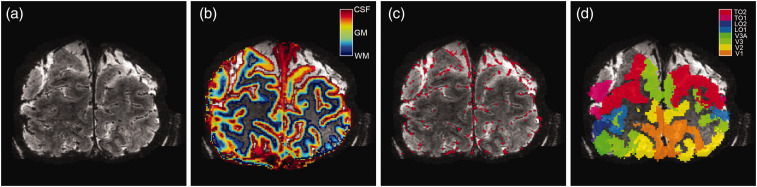
Volumetric maps. For 1 participant (subj09) the T2*-weighted anatomical volume (a), the layers segmentation (b), the pial vein estimation (c), and the visual area ROIs (d) are shown. Of the visual area ROIs, only V1, V2, and V3 were included.

The T1-weighted volume was divided by the PD volume to correct for large-scale intensity inhomogeneities.^36^ Afterwards, the T1-weighted volume was resampled to a resolution of 0.2 mm^3^ isotropic voxel size to estimate cortical layers at high spatial resolution (Figure 2). The cortex was divided into 20 equivolumetric layers using the LayNii software package.^37^ The term ‘layers’ should not be taken to represent architectonic layers distinguishable with histology. Here, ‘layers’ merely reflect discrete cortical depth levels.

The three T2*-weighted volumes were first realigned and averaged to increase SNR. The mean T2*-weighted volume was used to segment large veins, which have a near-zero intensity due to the low T2* value of blood and, therefore, appear black within the volume (Figure 2(a)). The vein segmentation was performed twice with different software packages that produced complementary results. First, large veins were estimated on the magnitude volume with Braincharter.^38^ Second, large veins were estimated again with Nighres^39^ on a quantitative susceptibility map (QSM). The QSM was reconstructed by Laplacian-based unwrapping and SHARP background filtering of the phase volume,^40,41^ and subsequently an iterative rapid two-step dipole inversion method.^42^ Both vein segmentation methods were combined by the union of the two separate vein masks to obtain the final estimated pial vein volume (Figure 2(c)).

A region-of-interest (ROI) approach was adopted, consisting of the primary visual cortex (V1) and extra-striate areas V2 and V3. Estimates of early visual cortical areas V1, V2, and V3 were constructed using a whole-brain 3 tesla T1-weighted volume that was available for the participants. A white and grey matter cortical surface was estimated on the 3T T1-weighted volume with Freesurfer (https://surfer.nmr.mgh.harvard.edu). The cortical surface reconstructions were then used to generate surface-based visual area maps using the anatomically defined Benson atlas of visual areas with Neuropythy (https://github.com/noahbenson/neuropythy).^43^ The visual area maps were projected back to volumetric space, and through a co-registration of 3T and 7T T1-weighted volumes using AFNI’s 3dAllineate, transformed to 7T T1-weighted space (Figure 2(d)).

All functional volumes were corrected for rigid body head motion with AFNI’s 3dvolreg (afni.nimh.nih.gov). The EPI geometric distortions were corrected using AFNI’s 3dQwarp. The EPI distortion correction and the motion correction were simultaneously applied in a single interpolation step using 3dNwarpApply to generate motion-corrected undistorted functional time series.^44,45^ An affine registration was then performed between the mean volume of the functional time-series and the T1-weighted anatomical volume using antsRegistration (http://stnava.github.io/ANTs/).^46^ The inverse of this transformation matrix was used to transform the previously computed cortical depth mask, pial vein mask, and V1, V2, V3 ROIs to the origin and dimensions of the functional volumes using a nearest-neighbor interpolation. Lastly, the time series were spatially smoothed using a Gaussian kernel with full-width-at-half-maximum (FWHM) = 2.35 mm, whilst being restricted to a cortical depth bin and ROI. Thus, spatial smoothing only was applied to voxels within the same cortical depth bin and the same ROI. Here, for spatial smoothing (and later statistical analyses), the 20 originally created equivolumetric layers were downscaled to 3 cortical depth bins (i.e., deep, middle, and superficial cortical layers) as follows: 18 cortical depth bins were selected by removal of the top and bottom layers levels (corresponding to white matter and cerebrospinal fluid (CSF) voxels), which were divided into 3 cortical depth bins, i.e., a down sampling of the remaining 18 cortical depth bins by a factor of 6. This smoothing procedure prevented the blurring of voxel data between different cortical depth levels or visual areas. The functional time series were then high-pass filtered using a discrete cosine transform filtering with a cut-off at 0.003 Hz and re-scaled to percent signal change (%BOLD) afterwards.

### FMRI data analysis

Estimates of the change in percent BOLD (%ΔBOLD) for each of the hypercapnia and hyperoxia levels were calculated using a general linear model (GLM). The GLM regressors consisted of a binary time series for each available breathing condition and a set of nuisance regressors consisting of 6 rigid-body head motion parameters and the RVT and BPM. We used binary regressors for the breathing conditions (i.e., value of 1 during the respective condition, 0 otherwise) in order to separate the connected hypercapnia conditions, and obtain regression coefficients for both. A second benefit of the binary gas condition regressors is that they are not affected by any residual transient signal changes (e.g. caused by movements), but rather fit the average plateau of the 2 minute hypercapnia and hyperoxia conditions. The regression coefficients during each breathing condition after significance thresholding (see statistical analysis) serve as %ΔBOLD for each voxel. The %ΔBOLD values for the hypercapnia conditions (i.e., %ΔBOLD_hc_) were then used to calculate CVR. For each voxel within participants, a linear regression was performed between the measured %ΔBOLD_hc_ and PetCO_2_ increase (i.e., ΔPetCO_2_ (mmHg)). The CVR, thus, represents the linear relationship of %BOLD and hypercapnic vasoactive stimulation for each voxel.^27,47–49^

To estimate the relative change in CBV, we first used the %ΔBOLD values from the hyperoxia condition (%ΔBOLD_ho_) to estimate the M-value using the hyperoxia-calibrated BOLD model from Chiarelli et al.:^50^

(1) $$\frac{\Delta \mathrm{BOLD}}{{\mathrm{BOLD}}_{0}}=M\cdot \left(1-\left(\frac{\mathrm{CBV}}{{\mathrm{CBV}}_{0}}\right)\left({\left(\frac{{\left[\mathrm{dHb}\right]}_{v}}{{\left[\mathrm{dHb}\right]}_{v0}}\right)}^{\beta }\right)\right)$$

Where M is a scaling parameter that represents the [dHb]-driven theoretical maximum signal change. The subscript “0” refers to baseline conditions, and the subscript “*v*” refers to venous properties. The “β” represents the influence of deoxygenated hemoglobin on transverse relaxation, and is estimated at *β* ≈ 1 for 7 tesla MRI.^51–53^ In the following equations we use β = 1, similar to a previous 7T laminar vascular space occupancy (VASO) MRI study.^24^ The change in CBV relates to the change in CBF following the Grubb’s law.^54^ Here, we assume a CBF/CBV coupling exponent *α* = 0.2, which has previously been estimated to reflect venous properties:^55^

(2) $$\left(\frac{\mathrm{CBV}}{{\mathrm{CBV}}_{0}}\right)={\left(\frac{\mathrm{CBF}}{{\mathrm{CBF}}_{0}}\right)}^{\alpha }$$

Because hyperoxia is generally believed to have a negligible effect on the change in CBF,^50^ equation (1) under hyperoxia conditions can be simplified to:

(3) $$\frac{\Delta \mathrm{BOLD}}{{\mathrm{BOLD}}_{0}}=M\cdot \left(1-{\left(\frac{{\left[\mathrm{dHb}\right]}_{v}}{{\left[\mathrm{dHb}\right]}_{v0}}\right)}^{\beta }\right)$$

This means that the change in BOLD signal under hyperoxia conditions is a function of the change in venous de-oxyhemoglobin concentration ([dHb]_v)_ and the M-value (scalar). The change in [dHb]_v_ can be estimated through standard formulas of oxygen transportation in the blood and by assuming a baseline oxygen extraction fraction (OEF). The M-value can be estimated by the following equation:^48,54,55^

(4) $${\;}^{49}M=\frac{\frac{\Delta \mathrm{BOLD}}{\mathrm{BOL}D_{0}}}{\left(1-{\left(\frac{F_{\left[\mathrm{dHb}\right]}}{F_{\left[\mathrm{dHb}\right]0}}\right)}^{\beta }\right)}$$

The relative change in fractional [dHb] (F_[dHb]_) during hyperoxia conditions represents the [dHb]_v_/[dHb]_v0_ ratio from equation (3). The equations necessary to calculate the fractional change in [dHb] are given in Supplementary Material II.

With the estimated M-value from the hyperoxia condition, we can estimate the change in venous CBV. The Davis model, which is particularly appropriate for hypercapnia conditions, describes the change in [dHb] as a function of the change in CMRO_2_ and CBF:^29,30^

(5) $$\frac{{\left[\mathrm{dHb}\right]}_{v}}{{\left[\mathrm{dHb}\right]}_{v0}}=\frac{{\mathrm{CMR}}_{O2}}{{\mathrm{CMR}}_{02|0}}\cdot \frac{{\mathrm{CBF}}_{0}}{\mathrm{CBF}}$$

Using equations (2) and (5), we can transform equation (1) to:

(6) $$\frac{\Delta \mathrm{BOLD}}{{\mathrm{BOLD}}_{0}}=M\cdot \left(1-{\left(\frac{\mathrm{CBV}}{{\mathrm{CBV}}_{0}}\right)}^{\frac{-\beta }{\alpha }}\cdot {\left(\frac{{\mathrm{CMR}}_{O2}}{{\mathrm{CMR}}_{02|0}}\right)}^{\beta }\right)$$

Hypercapnia conditions cause a small metabolic decrease, and was previously estimated to be approximately a 15% decrease for 90% CBF increase (+22 mmHg CO_2_).^57^ The effect is believed to scale linearly with CBF increase (and therefore with CO_2_ inspiration), which is why we adopt the following values for $\left(\frac{{\mathrm{CMR}}_{O2}}{{\mathrm{CMR}}_{02|0}}\right)$ during +3 mmHg, +5 mmHg, +8 mmHg, and +10 mmHg PetCO_2_: [0.97; 0.95; 0.92; 0.90], respectively.^56^ Now with the estimated M-value from the hyperoxia condition, we can estimate the change in CBV relative to baseline (i.e., *CBV/CBV_0_* = *ΔCBV*) as follows:

(7) $$\frac{\mathrm{CBV}}{{\mathrm{CBV}}_{0}}=\left(-{\left(\frac{\Delta \mathrm{BOLD}/{\mathrm{BOLD}}_{0}}{M}-1\right)}^{\frac{-\beta }{a}}/{\left(\frac{{\mathrm{CMR}}_{O2}}{{\mathrm{CMR}}_{02|0}}\right)}^{1-\alpha }\right)$$

### Statistical analysis

A GLM was constructed that consisted of gas challenge regressors and nuisance regressors (i.e., motion & physiology parameters). Hypercapnia and hyperoxia condition t-statistics were calculated on the basis of regression coefficients for the individual gas challenges. Only voxels that responded significantly to the gas-challenges were selected for further analyses (p < 0.05, Holm-Bonferroni corrected). Additional masks were imposed by the cortical depth and ROI mask, thereby including only those voxels that were in range of grey matter cortical layers within visual areas V1, V2, and V3.

Separate linear mixed models (LMM) were constructed with “%ΔBOLD_hc_”, “%ΔBOLD_ho_”, “CVR”, “M”, “ΔCBV” as dependent variables, and with the participants as a random-effects grouping factor. The usage of an LMM analysis allows for the inclusion of each voxel as a separate observation for each of the metrics. Additionally, the model is capable of handling missing values for +3 mmHg and +8 mmHg PetCO_2_ hypercapnia levels. Therefore, all conditions and measurements of all participants can be included. Each LMM had the following ‘fixed effects’ variables: scan sequence (i.e., GE, SE), and cortical depth (i.e., values ranging from 1 to 3 for each cortical depth bin). The LMM for %ΔBOLD_hc_ and ΔCBV, additionally, have the measured PetCO_2_ as a fixed effect variable. Please note, that the significance test for the effect of PetCO_2_ on %ΔBOLD_hc_ and the test for CVR are a closely related, but not identical: if all hypercapnia levels cause a similar increase in %ΔBOLD_hc_, this may result in a significant effect of PetCO_2_ on %ΔBOLD_hc_, whereas the CVR (i.e., the slope of ΔPetCO_2_ and %ΔBOLD_hc_) will then be zero and, therefore, not significant. Random slopes of the LMMs were estimated across the participant random effect. The LMMs were fitted using the restricted maximum likelihood (REML) approach, and the degrees of freedom were calculated using the Satterthwaite model. The statistical tests were performed using *JASP* (V.0.15, www.jasp-stats.org).

## Results

### GE & SE %ΔBOLD for vasoactive stimuli across cortical depth

**Figure 3.**
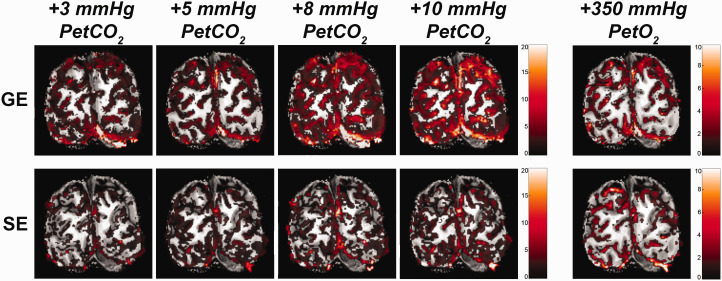
Volumetric BOLD effect from vasoactive stimuli. The %ΔBOLD is shown for one participant (subj08) for the 4 hypercapnia levels: +3, +5, +8, +10 mmHg PetCO_2_, and +350 mmHg PetO_2_, as measured with GE (top panels) and SE (bottom panels).

**Figure 4.**
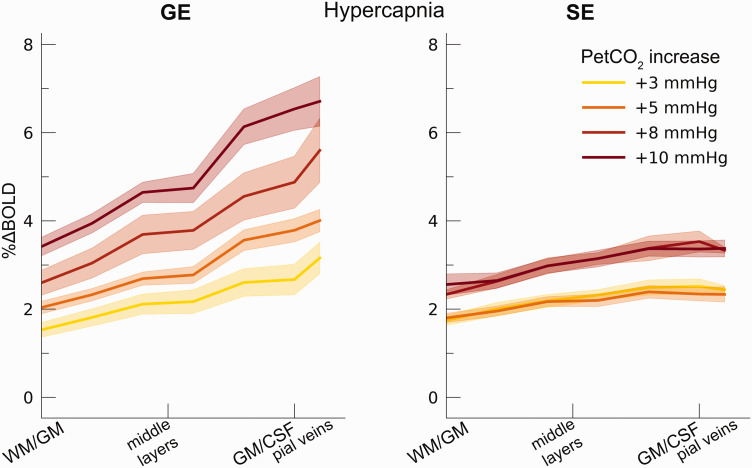
Percent BOLD signal change across cortical depth during hypercapnia. The %ΔBOLD_hc_ is shown across cortical depth (x-axis) for the 4 hypercapnia levels (colors) and GE vs SE scan sequences (left/right panels). The shaded area represents the SEM across participants.

We observed an average increase in GE and SE BOLD percent signal change following the hypercapnia conditions (i.e., +3 mmHg, +5 mmHg, +8 mmHg, and +10 mmHg: F_(1,9.9)_ = 54.17, p < .001). The %ΔBOLD_hc_ differed significantly between GE and SE (F_(1,9.9)_ = 5.40, p = .043. Figure 3), indicating that the measurements from all venous vessels produced on average significantly different BOLD signal amplitudes compared to measurements from the micro-vasculature (mean %ΔBOLD_hc_ GE = 3.85%, 95% CI = [3.58%, 4.12%]; mean %ΔBOLD_hc_ SE = 2.62%, 95% CI = [2.39%, 2.84%]). We find a strong interaction effect of %ΔBOLD_hc_ with cortical depth (F_(1,10.0)_ = 29.06, p < .001) and a significant three-way interaction of %ΔBOLD_hc_, cortical depth and scan sequence (F_(1,10.4)_ = 20.32, p = .001). These results indicate that %ΔBOLD_hc_ increases more strongly with increased CO_2_ inspiration at superficial layers than deeper layers. This effect was prominent for GE BOLD as opposed to SE BOLD (Figure 4).

**Figure 5.**
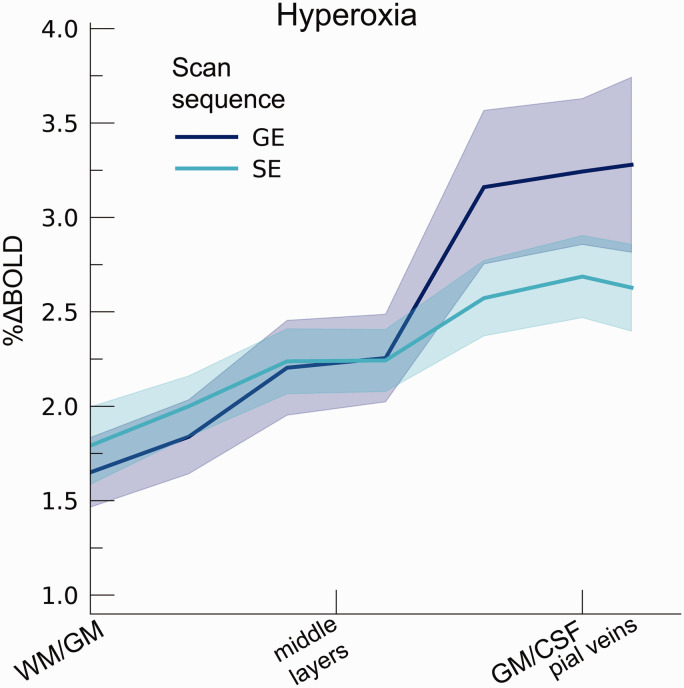
Percent BOLD signal change across cortical depth during hyperoxia. The %ΔBOLD_ho_ is shown across cortical depth (x-axis) for GE and SE sequences (colors). The shaded area represents the SEM across participants.

The hyperoxia condition also increased %ΔBOLD_ho_ (t_(7.7)_ = 8.97, p < .001; mean %ΔBOLD_ho_ GE = 2.48%, 95% CI = [1.89%, 3.07%]; mean %ΔBOLD_ho_ SE = 2.28%, 95% CI = [1.92%, 2.64%]). In contrast to the hypercapnia conditions, %ΔBOLD_ho_ increased from deeper to superficial layers during both GE and SE BOLD acquisitions (F_(1,9.7)_ = 68.72, p < .001), without detecting a difference between scan sequences (F_(1,9.6)_ = 3.90, p = .078), nor an interaction of scan sequence and cortical depth (F_(1,9.2)_ = 5.01, p = .052). These results signify that BOLD responses originating from macro- and micro-vascular compartments alike are all sensitive to the relative increase in [Hb_v_], having the largest effect near the pial surface (Figure 5).

### CVR across cortical depth and vascular compartments

**Figure 6.**
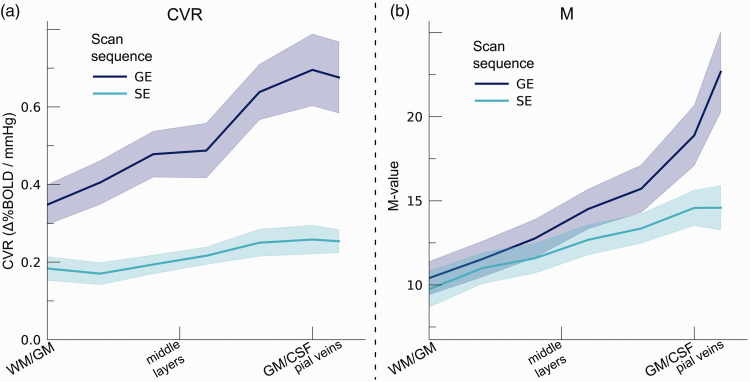
(a) CVR. The CVR is shown across cortical depth for the 2 scan sequences (colors) and (b) M-value. The theoretical maximum signal intensity “M-value” is shown across cortical depth for the 2 scan sequences (colors). The shaded area represents the SEM across participants.

CVR changed significantly across cortical depth. Generally, CVR increased towards the superficial layers (F_(1,10.0)_ = 34.16, p < .001). This increase was particularly apparent for GE BOLD, as shown by the significant interaction effect between scan sequence and cortical depth (F_(1,10.0)_ = 17.02 p = .002). In contrast, SE BOLD exhibited a small increase in CVR from deeper to superficial layers (Figure 6(a)). The deeper cortical layers showed an average estimate of CVR = 0.39%ΔBOLD/mmHg for GE (95% CI = [0.28%ΔBOLD/mmHg, 0.50%ΔBOLD/mmHg]) and CVR = 0.18%ΔBOLD/mmHg for SE (95% CI = [0.13%ΔBOLD/mmHg, 0.23%ΔBOLD/mmHg]), whereas at superficial cortical layers values were CVR = 0.64%ΔBOLD/mmHg for GE (95% CI = [0.50%ΔBOLD/mmHg, 0.79%ΔBOLD/mmHg]) and CVR = 0.25%ΔBOLD/mmHg for SE (95% CI = [0.20%ΔBOLD/mmHg, 0.31%ΔBOLD/mmHg]). Thus, the CVR across cortical depth derived from all venous vessels is over a factor of 2.5 larger compared to CVR from the micro-vasculature only.

### M-value across cortical depth and vascular compartments

We estimated the M-value based on the %ΔBOLD_ho_ and the PetO_2_ trace during hyperoxia (Figure 6(b)). The M-value increased strongly across cortical depth, peaking near the border of grey matter (GM) and CSF (F_(1,10.0)_ = 75.79, p < .001). However, a difference between GE and SE was not observed (F_(1,9.9)_ = 0.54, p = .479). M-value estimates reveal a substantial maximum signal change capacity for both scan sequences (mean M-value GE = 14.28%, 95% CI = [11.87%, 16.69%]; mean M-value SE = 12.26%, 95% CI = [10.58, 13.94]). We, additionally, observed an interaction effect of scan sequence and cortical depth (F_(1,9.8)_ = 9.88, p = .011). This interaction effect reflects the fact that no difference in M-value at deeper cortical layers between GE and SE sequences was observed (mean M-value deeper layers GE = 10.74%, 95% CI = [8.84, 12.65]; SE = 10.38%, 95% CI = [8.64, 12.62]; post-hoc z = 0.45, p = .653), while the M-value was significantly larger at superficial layers for GE compared to SE (mean M-value deeper layers GE = 17.81, 95% CI = [14.73, 20.88]; SE = 14.14, 95% CI = [12.22, 16.05]; post-hoc z = 2.97, p = .009).

### Hypercapnic ΔCBV across cortical depth and vascular compartments

**Figure 7.**
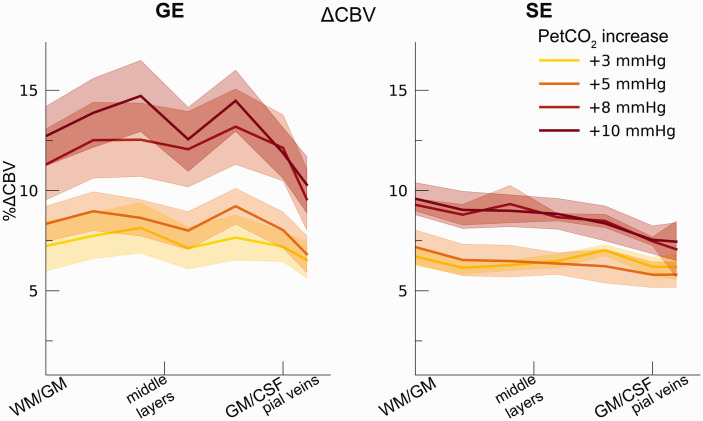
ΔCBV. The ΔCBV in percentages is shown across cortical depth for the 4 hypercapnia levels (colors) and GE/SE BOLD (left/right panels). The shaded area represents the SEM across participants.

Our ΔCBV estimate shows a significant effect of different hypercapnia levels (F_(1,10.2)_ = 28.63, p < .001), which is representative of an increase in ΔCBV with increased levels of inspired CO_2_ (Figure 7). We, additionally, observed an interaction effect of hypercapnia levels with the scan sequence (F_(1,9.3)_ = 8.61, p = .016) as the ΔCBV increase was approximately 1.35 times larger for GE (mean %ΔCBV = 10.1%, 95% CI = [8.1%, 12.2%]), compared to SE (mean %ΔCBV = 7.4%, 95% CI = [6.5%, 8.2%]). We did not observe a difference in ΔCBV across cortical depth (F_(1,15.5)_ = 0.23, p = .637). Thus, even though the different hypercapnia levels led to a gradual increase in CBV, this relative increase was approximately uniform across cortical depth for both GE and SE BOLD.

## Discussion

### General discussion

In the current study, we quantify the effects that different vascular compartments have on laminar BOLD fMRI signals using hypercapnic and hyperoxic stimuli. We find that increasing levels of hypercapnia result in increasing percent signal changes for both the scans that are sensitive to all venous vessels (GE BOLD) and the scans that are predominantly sensitive to the micro-vasculature (SE BOLD). However, the magnitude of the hypercapnia effect on the BOLD fMRI signal is strongly dependent on the vascular compartment from which the signal originates in combination with cortical depth level. This effect is signified by the increasing CVR across cortical depth as sampled from all venous vessels. CVR estimates from the micro-vasculature do not show substantial differences in vessel dilation capacity across cortical depth. The GE BOLD CVR estimates were approximately 2.5× larger compared to SE BOLD CVR estimates. The hyperoxia condition also leads to an increase in percent signal change, which together with the PetO_2_ allows for an estimation of the maximum theoretical BOLD signal: M-value. On the basis of the M-value, we find that both macro- and micro-vascular compartments are capable of comparable theoretical maximum signal intensities. The M-values increased clearly from deeper to superficial layers irrespective of vessel size, albeit that this trend was steeper, when BOLD signals also originated from the macro-vasculature: from deep to superficial layers the percent signal change ranged from 9 to 21 percent for all venous vessels and 9 to 16 percent for the micro-vasculature. Finally, we observed that increased levels of hypercapnia led to an increase in ΔCBV, which is significantly larger for BOLD signals derived from all venous vessels, as compared to micro-vasculature signals only (by a factor of 1.35), albeit that ΔCBV was distributed approximately uniformly across cortical depth in all measured vascular compartments.

#### Comparable M-values for all vascular compartments

We observed a mean increase in percent BOLD signal change following the hyperoxia condition of +350 mmHg PetO_2_ increase, in line with previous hyperoxia reports.^50,57–60^ The increase in PetO_2_ presented here equates to an air mixture consisting of roughly 60% O_2_, which is considered mild hyperoxia.^61,62^ It has previously been reported that mild cases of hyperoxia have a negligible effect on CBF.^57,60,63^ This assumption allows for the estimation of the theoretical maximal percent signal change (M-value) per voxel on the basis of the hyperoxia BOLD signal change and the measured PetO_2_ values. We find that larger and smaller vascular compartments alike are capable of generating comparable BOLD signal changes, purely on the basis of relative venous [Hb] increase. This resemblance was particularly observed for deep and middle cortical layers, while superficial cortical layers showed larger M-values, when measurements included macro-vascular signals (see also Figure 6(b)). On average, we find that the theoretical maximal BOLD signal change is lower at the deeper compared to the superficial cortical layers, ranging from 9 (deep) to 21 (superficial) percent signal change for all venous vessels and 9 (deep) to 16 (superficial) percent signal change for the micro-vasculature in line with previous high-field M-value estimations.^24,52,64^ The M-value increase with cortical depth is logically reconcilable with GE BOLD, since GE scans are relatively sensitive to larger veins and venules around the pial surface. Therefore, increased levels of (baseline) CBV that co-occur with larger vessels near the pial surface would lead to increased M-values for GE BOLD.^65^ However, the increase in M-value across cortical depth was also seen for SE BOLD, albeit with a smaller slope. One possible explanation for this observation may be that SE becomes sensitive to venules during hyperoxia, and the M-value trend reflects the higher density of venules toward the pial surface.^11,15^ Future studies could address the change in M-value as a function of distance from larger (pial) veins. Alternatively, it could be that the venous [Hb] increase in the micro-vasculature differs across cortical depth, although the physiological basis for this to occur is unclear. The need for validation of underlying physiological parameters is further emphasized by the assumptions made in current models. For M-value estimation, we assumed values for OEF, transverse relaxation parameter *β*, and CBF/CBV coupling exponent *α*. Due to these parameter assumptions, estimating the M-value per voxel is inherently a noisy process. Possibly, values for *α* and *β* differ between differently sized vascular compartments (and thereby scan sequence) and may, additionally, vary across cortical depth.^66,67^ Additional research is necessary to link these parameters to vascular compartment size. Given the current parameter assumptions, we find that the M-values for GE and SE BOLD were on the same order from deep to middle layers, indicating that GE BOLD is weighted toward the micro-vasculature (capillaries and possibly smaller venules) at these cortical depths.

#### CVR from all venous vessels is 2.5x larger than micro-vascular CVR

CVR is commonly used to describe vessel dilation properties.^26^ Here we show that all venous vessels combined as measured by GE BOLD have a greater capacity for vessel dilation than the micro-vasculature as measured by SE BOLD. We observed that the CVR increases for GE BOLD from deeper towards superficial cortical layers, likely reflecting the increasing venous macro-vascular density toward the pial surface and potentially a corresponding increased reactivity.^11,15^ This effect was not observed for the micro-vasculature, which has been reported capable of dilation.^68,69^ These findings, however, indicate that capillaries and possibly smaller venules and arterioles have a smaller capacity for dilation as compared to all venous vessels, reflected by an approximate 2.5 fold CVR increase (CVR GE/SE ratio ranges from 2.2 (deep) to 2.6 (superficial)). Additionally, the current findings suggest that neuronal signals as conveyed by the neurovascular coupling from smaller vascular compartments are limited by the maximum dilation capacity of capillaries.^22^ The M-value, however, indicates that the micro-vasculature is capable of generating BOLD signal changes comparable to larger vessels in a considerable portion across the depth of cortex. The fact that large BOLD signals from smaller vascular compartments are not frequently observed,^14,70^ could perhaps stem from the inability of the smallest vessels to dilate substantially. Since CVR is often interpreted as a proxy for vessel health, high-spatial resolution vessel health measurements based on CVR should account for different dilation properties of differently sized vascular compartments that do not directly relate to the healthiness of the vasculature across cortical depth. Moreover, CVR measurements obtained with SE BOLD might lead to better predictors of small vessel disease^71,72^ than CVR measurements with GE BOLD. SE BOLD CVR can be used to measure potential micro-vascular hemodynamic impairment with the macro-vasculature having less of an impact on CVR measurements. However, CVR values estimated from SE BOLD are substantially smaller compared to CVR values derived from GE BOLD. It may, therefore, be more challenging to reliably estimate CVR values from SE BOLD. Additionally, we found that on the basis of visual inspection, SE BOLD does not respond in a similar fashion to different hypercapnic levels compared to GE BOLD. Namely, the lower PetCO_2_ increase conditions (i.e., +3 mmHg and +5 mmHg) resulted in roughly equal SE BOLD signal changes, as did the higher PetCO_2_ increase conditions (i.e., +8 mmHg and +10 mmHg, see also Figure 4). The micro-vasculature reactivity response profile to different inspired PetCO_2_ levels might not be as diverse as is observed with GE BOLD. Here, it is worth considering that GE and SE scans represent different contrasts (i.e., T2* and T2, respectively). The refocusing of spins, creating the T2 SE BOLD contrast, leads to smaller signal amplitude changes compared to GE BOLD contrasts within the same micro-vascular compartments,^73^ which could potentially bias current CVR estimates of both contrast mechanisms. Furthermore, GE and SE contrasts may also be influenced under hypercapnia conditions by baseline OEF, baseline CBV, frequency offset for fully deoxygenated blood and the changes in [dHb] within the probed vessels.^73,74^ Further studies and simulation work are necessary to quantify their respective contributions.

### ΔCBV uniformly distributed across cortical depth

Through the measurements of BOLD signal change during hypercapnia levels, the PetCO_2_ trace, and the estimation of the M-value, we have been able to estimate the change in CBV relative to baseline venous CBV. A clear increase in ΔCBV is seen for increasing levels of inspired CO_2_, which causes vessels to dilate. An increase of 12.5% ΔCBV is seen during the highest hypercapnia level (i.e., +10 mmHg PetCO_2_), when measured from all venous vessels. The same hypercapnia level as measured from the micro-vasculature causes on average 8.5% ΔCBV change. Contrary to the other metrics of the current study, we find no significant difference in ΔCBV across cortical depth for any of the vascular compartments. However, a small dip in ΔCBV around the middle cortical layers can be observed (Figure 5), which has previously been observed with direct ΔCBV measurements, albeit with different experiment conditions.^4,75^ Possibly, the absence of clear ΔCBV changes across cortical depth is indicative of a conservation of matter, i.e., blood volume in this case. This suggests that in all cortical layers a comparable relative ΔCBV increase can be expected in early visual cortex following vasoactive stimuli or neurovascular stimuli of extended duration,^4^ albeit that the absolute change in CBV likely scales with vessel density and diameter. Lastly, the ΔCBV estimation is established through M-value estimation and is, therefore, similarly affected by parameter assumptions as the estimated M-values plus additional assumptions on the change in CMRO_2_ following hypercapnic breathing conditions. Reported results are, thus, likely dependent on these assumed parameter values.

### Experimental considerations

There were several factors in the experimental design that may have influenced our results. First, we have not been able to obtain all four hypercapnia levels (i.e., +3 mmHg, +5 mmHg, +8 mmHg, and +10 mmHg PetCO_2_) in all participants. All participants have engaged in the +5 mmHg and +10 mmHg PetCO_2_ breathing challenges, therefore current results of CVR and ΔCBV may be skewed towards these conditions. However, missing values were dealt with by employing LMMs for statistical modeling, thereby including all available observations of the petCO_2_ and estimating random slopes per participant. A second experimental factor concerns the spatial resolution of the SE scan sequence, which entailed a 1.5 mm isotropic voxel size. This spatial resolution was selected to attain a sufficiently large SNR, but simultaneously increases partial volume effects that can result in a blurring of cortical layers and possibly the inclusion of white matter and CSF signals. Therefore, the presented differences between different vascular compartments may be influenced by the acquired voxel size.

### Conclusions

Laminar BOLD fMRI is affected by vascularization differences that exist across cortical depth. In the current study, we show that vascular compartments of all sizes are capable of generating comparable percent BOLD signal changes across cortical depth. However, the maximum BOLD signal change from the micro-vasculature may be limited by the extend of the microvascular capacity to dilate, as represented by the CVR values. BOLD signals that are dominated by larger vessels showed 2.2 to 2.6 times larger CVR values in comparison to the microvasculature CVR values across cortical depth. Additionally, the relative change in CBV was 1.35 times larger when measured from all venous vessels, compared to the micro-vasculature only. This finding was not dependent on cortical depth, indicating that the change in CBV is not relatively larger for pial draining veins compared to smaller vessels in early visual cortex. The current study offers novel estimations on the origin and magnitude of BOLD fMRI signal changes. This allows future laminar BOLD fMRI studies to account and possibly correct for signal changes in relation to the vascular compartments from which the signals originate.

## Supplemental Material

sj-pdf-1-jcb-10.1177_0271678X221133972 - Supplemental material for The many layers of BOLD. The effect of hypercapnic and hyperoxic stimuli on macro- and micro-vascular compartments quantified by *CVR*, *M*, and *CBV* across cortical depth Click here for additional data file. Supplemental material, sj-pdf-1-jcb-10.1177_0271678X221133972 for The many layers of BOLD. The effect of hypercapnic and hyperoxic stimuli on macro- and micro-vascular compartments quantified by *CVR*, *M*, and *CBV* across cortical depth by Wouter Schellekens, Alex A Bhogal, Emiel CA Roefs, Mario G Báez-Yáñez, Jeroen CW Siero, Natalia Petridou in Journal of Cerebral Blood Flow & Metabolism

sj-pdf-2-jcb-10.1177_0271678X221133972 - Supplemental material for The many layers of BOLD. The effect of hypercapnic and hyperoxic stimuli on macro- and micro-vascular compartments quantified by *CVR*, *M*, and *CBV* across cortical depth Click here for additional data file. Supplemental material, sj-pdf-2-jcb-10.1177_0271678X221133972 for The many layers of BOLD. The effect of hypercapnic and hyperoxic stimuli on macro- and micro-vascular compartments quantified by *CVR*, *M*, and *CBV* across cortical depth by Wouter Schellekens, Alex A Bhogal, Emiel CA Roefs, Mario G Báez-Yáñez, Jeroen CW Siero, Natalia Petridou in Journal of Cerebral Blood Flow & Metabolism

## Data Availability

All data will be accessible through Flywheel.
