# Supplementary material for: The many layers of BOLD. The effect of hypercapnic and hyperoxic stimuli on macro- and micro-vascular compartments quantified by CVR, M, and CBV across cortical depth
Source: J Cereb Blood Flow Metab. 2022 Oct 19;43(3):419–32. doi: 10.1177/0271678X221133972 (PMC9941862; doi:10.1177/0271678X221133972)
Supplement: sj-pdf-1-jcb-10.1177_0271678X221133972 - Supplemental material for The many layers of BOLD. The effect of hypercapnic and hyperoxic stimuli on macro- and micro-vascular compartments quantified by CVR, M, and CBV across cortical depth [file sj-pdf-1-jcb-10.1177_0271678X221133972.pdf]

	+3 mmHg CO_2		+5 mmHg CO_2		+8 mmHg CO_2		+10 mmHg CO_2		+350 mmHg O_2	
	**GE**	**SE**	**GE**	**SE**	**GE**	**SE**	**GE**	**SE**	**GE**	**SE**
**Sub01**	.	.	3.8	4.1	.	.	8.4	8.0	374.6	373.4
**Sub02**	.	.	2.7	2.2	.	.	7.4	7.7	369.0	366.8
**Sub03**	.	.	4.4	4.2	.	.	7.4	7.9	349.3	332.3
**Sub04**	2.7	.	5.1	5.2	5.5	.	9.0	8.4	276.4	294.1
**Sub05**	.	3.3	5.7	4.9	.	7.3	10.3	9.4	324.0	318.8
**Sub06**	.	.	4.5	4.9	.	.	8.7	8.4	358.3	342.1
**Sub07**	.	3.6	5.8	5.1	.	6.8	8.9	8.1	357.4	334.7
**Sub08**	4.1	3.9	6.3	6.2	8.2	8.0	10.2	10.3	345.1	332.2
**Sub09**	3.7	3.7	5.8	5.7	7.2	7.4	10.0	9.5	368.7	362.6
**Sub10**	3.7	3.4	5.0	5.3	7.2	7.0	8.5	8.7	309.4	316.7
**Sub11**	2.8	2.5	4.3	4.3	6.1	6.0	7.8	8.0	273.3	278.3

TABLE CAPTION

Supplementary material I. Average obtained $PetCO_2$ and $PetO_2$ values. For each of the 4 targeted $PetCO_2$ values (+3, +5, +8, +10 mmHg PetCO2), during GE and SE BOLD acquisition, the average obtained $PetCO_2$ values are shown (columns) for each participant (rows). The last 2 columns show the average obtained $PetO_2$ values for each participant.
