# Supplementary material for: The many layers of BOLD. The effect of hypercapnic and hyperoxic stimuli on macro- and micro-vascular compartments quantified by CVR, M, and CBV across cortical depth
Source: J Cereb Blood Flow Metab. 2022 Oct 19;43(3):419–32. doi: 10.1177/0271678X221133972 (PMC9941862; doi:10.1177/0271678X221133972)
Supplement: sj-pdf-2-jcb-10.1177_0271678X221133972 - Supplemental material for The many layers of BOLD. The effect of hypercapnic and hyperoxic stimuli on macro- and micro-vascular compartments quantified by CVR, M, and CBV across cortical depth [file sj-pdf-2-jcb-10.1177_0271678X221133972.pdf]

The venous deoxygenated fraction of [Hb] is calculated as described by Chiarelli et al. (2009) [46], using the Severinghaus equation [52]:

$$Sa_{O_2} = \frac{1}{\left(\frac{23400}{\left(Pa_{O2}{}^{3}\right)+150\left(Pa_{O2}\right)}+1\right)} \qquad (1)$$

, where the measured $PetO_2$ values can be used for the Pa_{O2} parameter. Next, the arterial oxygen content (Ca_{O2}) can be estimated by assuming literature standard values for: the O_2-carrying capacity of hemoglobin (φ: 1.34 ml O_2 / g_{hb} in humans), the concentration of hemoglobin ([Hb]: 15 g Hb / dl blood), and the solubility coefficient of oxygen in blood (ε: 0.0031 ml O_2 / (dl blood * mmHg):

$$Ca_{O2} = (\varphi \cdot [Hb] \cdot Sa_{O2}) + (Pa_{O2} \cdot \varepsilon) \qquad (2)$$

The venous oxygen content (Cv_{O2}) depends on the Ca_{O2} and the OEF:

$$Cv_{O2} = Ca_{O2} - (Ca_{O2}|_0 \cdot OEF) \qquad (3)$$

In equation (3), we assume a baseline OEF = 0.3 [46,51]. The fractional venous oxygen saturation (Sv_{O2}) can then be estimated as follows:

$$Sv_{O2} = \frac{Cv_{O2} - (Pv_{O2} \cdot \varepsilon)}{\varphi \cdot [Hb]} \qquad (4)$$

In equation (4), the Pv_{O2} represents the oxygen dissolved in venous plasma and is believed to have a negligible small effect[46] (i.e., $Pv_{O2} \approx 0$). At this point, we can estimate the deoxygenated fraction of [Hb] ($F_{[dHb]}$) from Sv_{O2}:

$$F_{[dHb]} = 1 - Sv_{O2} \qquad (5)$$

SUPPLEMENTARY TEXT CAPTION

Supplementary material II. Fractional [dHb] calculation.
